# Electroporating Fields Target Oxidatively Damaged Areas in the Cell Membrane

**DOI:** 10.1371/journal.pone.0007966

**Published:** 2009-11-23

**Authors:** P. Thomas Vernier, Zachary A. Levine, Yu-Hsuan Wu, Vanessa Joubert, Matthew J. Ziegler, Lluis M. Mir, D. Peter Tieleman

**Affiliations:** 1 Ming Hsieh Department of Electrical Engineering, Viterbi School of Engineering, University of Southern California, Los Angeles, California, United States of America; 2 MOSIS, Information Sciences Institute, Viterbi School of Engineering, University of Southern California, Los Angeles, California, United States of America; 3 Department of Physics and Astronomy, University of Southern California, Los Angeles, California, United States of America; 4 Mork Family Department of Chemical Engineering and Materials Science, Viterbi School of Engineering, University of Southern California, Los Angeles, California, United States of America; 5 Centre National de la Recherche Scientifique, Unité Mixte de Recherche 8121–Vectorology and Gene Transfer, Institut Gustave Roussy, Villejuif, France; 6 Unité Mixte de Recherche 8121, Université Paris-Sud, Orsay, France; 7 Department of Biological Sciences, University of Calgary, Calgary, Alberta, Canada; Hebrew University of Jerusalem and University of California, Berkeley, Israel

## Abstract

Reversible electropermeabilization (electroporation) is widely used to facilitate the introduction of genetic material and pharmaceutical agents into living cells. Although considerable knowledge has been gained from the study of real and simulated model membranes in electric fields, efforts to optimize electroporation protocols are limited by a lack of detailed understanding of the molecular basis for the electropermeabilization of the complex biomolecular assembly that forms the plasma membrane. We show here, with results from both molecular dynamics simulations and experiments with living cells, that the oxidation of membrane components enhances the susceptibility of the membrane to electropermeabilization. Manipulation of the level of oxidative stress in cell suspensions and in tissues may lead to more efficient permeabilization procedures in the laboratory and in clinical applications such as electrochemotherapy and electrotransfection-mediated gene therapy.

## Introduction

External electric fields of sufficient strength and duration cause a rapid increase in the electrical conductance of biological membranes, with an associated increased permeability to ions and small and large molecules [1]–[3]. If the dose is limited, cells can survive the treatment. Electropermeabilization (also called electroporation) technology is widely used in laboratories to facilitate transfection in cells and tissues [4]–[6] and recently has appeared in the clinic as a component of systems for electrochemotherapy [7] and tumor killing and ablation [8]–[11]. Although the physical and electrochemical fundamentals of electric field-induced permeabilization of lipid membranes are well known [12]–[14], the mechanistic details of the membrane restructuring that follows electric field exposure in living cells have not been definitively established. Electrical measurements [15], flow cytometry [16], and fluorescence microscopy [17] indicate that permeabilization can occur in less than 10 ns, implying a direct rearrangement of membrane components, but real-time analysis of any kind at this time scale is difficult [18]–[19]. Observations with living cells [20], artificial membranes and lipid vesicles [21]–[24], continuum electrophysical models [25]–[26], and molecular dynamics (MD) simulations of phospholipid bilayers [27]–[31] provide a valuable perspective, but significant gaps remain between these model systems and the complexity of the tissue in an electropermeabilized tumor.

Optimizing the efficiency of electroporation methods requires, in addition to knowledge of the effects of varying the relevant physical parameters (electric field strength and duration, temperature, cell concentration, composition of the medium, electrode arrangement) [32]–[34], an understanding of the relation between the physiological state of cells and their susceptibility to electropermeabilization. It may be, for example, that starved cells can be most effectively treated under conditions that are very different from those that are optimal for actively respiring cells.

Because oxidative stress is readily imposed and commonly encountered in cultured cells and in whole organisms under a variety of conditions, because it impacts cellular activities across the metabolic spectrum, and because it directly affects the physical properties of the cell membrane [35]–[37], we speculated that this might be an important factor in the effectiveness of electroporation methods. Studies of the peroxidation of membrane lipids *after* electroporation have been reported [38]–[41], but the effects of pre-treatment oxidative stress have received little experimental attention, and so we investigated the effect of membrane oxidative damage on the sensitivity of cells to permeabilizing electrical pulses. Molecular dynamics simulations have recently shown that incorporating oxidized lipids into phospholipid bilayers increases the water permeability of these membranes [42], suggesting that bilayers containing oxidized lipids will also electroporate more readily (the formation of membrane-spanning water defects is one of the initial steps in molecular dynamics representations of electroporation). That is the hypothesis that we tested in the work reported here, in simulations and in living cells.

## Results

### Molecular Dynamics Simulations

#### Pore formation time for PLPC and oxidized PLPC bilayers

**Figure 1:**
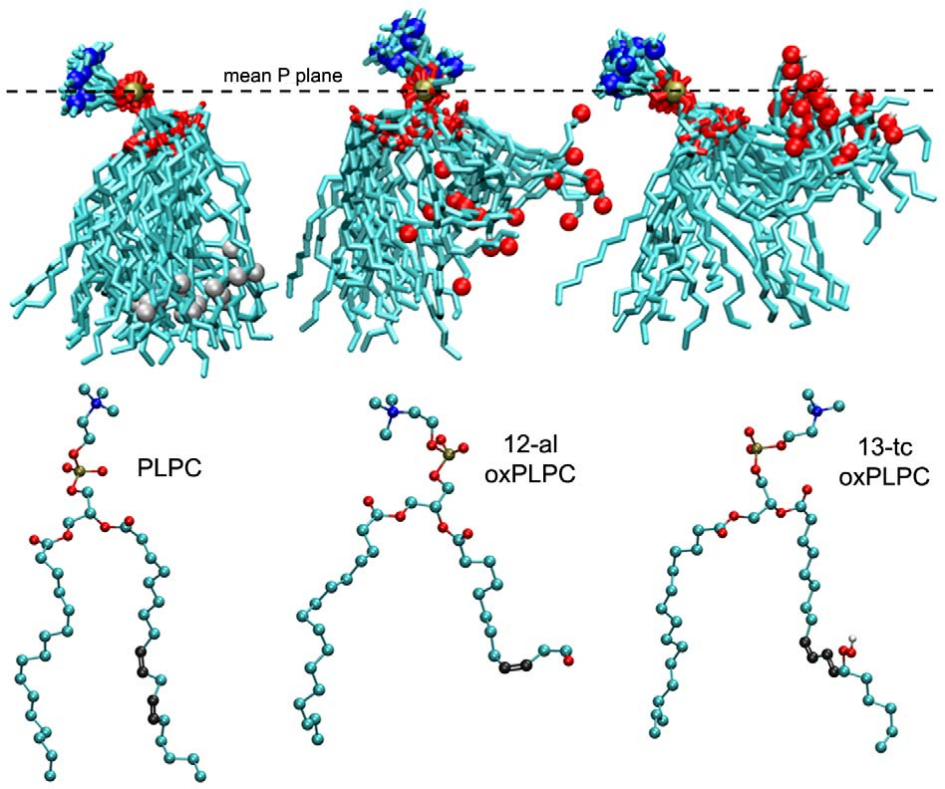
Oxidized and unoxidized phospholipid conformations change over time. Composite snapshots (21 images captured at 0.5 ns intervals over a 10 ns period) of PLPC and two oxidized variants, oxPLPC (12-al) and oxPLPC (13-tc), showing their conformations in molecular dynamics simulations of PLPC with 11% oxPLPC bilayers in a 360 mV/nm field. The spheres near the end of the lipid tails mark the location of the introduced oxygens or C-13 of PLPC. Structures of the individual lipid molecules are shown below the corresponding composite. Teal – C, red – O, gold – P, blue – N, gray – C-13.

Two oxidized variants of 1-palmitoyl-2-linoleoyl-*sn*-glycero-3-phosphatidylcholine (PLPC) were chosen for this study based on the increased water permeability of PLPC bilayers containing these species in molecular dynamics simulations [42]. Construction of the oxidized PLPC (oxPLPC) molecules containing the modified linoleoyl residues 12-oxo-*cis*-9-dodecenoate (12-al) and 13-hydroperoxy-*trans*-11, *cis*-9-octadecadienoate (13-tc) has been previously described [42]. Composite snapshots of PLPC and the 12-al and 13-tc variants taken from the simulations with external electric fields reported here (Figure 1) show the location of the oxidized molecular modifications and how the conformation of the oxidized lipid tails contributes to an increase in area per lipid when these species are incorporated into a PLPC bilayer (Table 1). The stabilization associated with hydration of the tail oxygens in the oxidized lipids results in a tendency for the oxidized tails to spend more time near the aqueous interface than the hydrocarbon tails of PLPC. In systems containing 11% oxPLPC the mean distance from the phosphorus plane (membrane interface region) to the C-12 oxygen atom in 12-al (0.6 nm) and to the C-13 oxygens in 13-tc (0.3 nm) is much less than that for the *sn*-2 C-13 in PLPC (1.6 nm).

**Table 1:** Effect of oxidized lipid concentration on electropore formation time in oxPLPC:PLPC bilayers.

oxPLPC		Thickness (nm)	Area/Lipid (nm^2^)	Trial	Poration Time (ns)	
					Individual	Mean (S.D.)
				1	>25	
**None**	0%	3.8	0.68	2	>25	>22.6
				3	22.6	
				1	16.5	
	11%	3.4	0.68	2	6.3	8.4±7.3
				3	2.3	
				1	12.5	
**12-al**	25%	3.5	0.69	2	6.2	7.6±4.4
				3	4.0	
				1	2.9	
	50%	3.3	0.72	2	1.1	1.8±1.0
				3	1.4	
				1	>55	
	11%	3.8	0.69	2	43.0	>22.9
				3	2.7	
				1	22.8	
**13-tc**	25%	3.7	0.70	2	13.4	14.4±7.9
				3	7.1	
				1	5.4	
	50%	3.6	0.72	2	4.9	4.7±0.9
				3	3.7	

Bilayer thickness (distance between the two mean phosphorus planes) and area per lipid are mean values taken from three independent simulations for each condition over 100 ps time steps from the beginning of the simulation until 1 ns before pore formation. For systems where poration occurred in less than 1.5 ns, the cutoff is 0.5 ns before pore formation. Bilayer thickness values from independent simulations vary by less than 10%. Area per lipid is the area of the bilayer divided by the number of lipids in one leaflet of the bilayer (36). Individual and mean times to poration are shown for three independent simulations of each system. Systems contain 2880 water molecules and 72 total lipids. The applied electric field is 360 mV/nm.

To observe the effect of oxidized lipids on the sensitivity of a bilayer to electroporating fields, equilibrated systems composed of 72 lipids and 2880 waters with varying percentages of PLPC and one of the two oxPLPC species were assembled and subjected to an external electric field of 360 mV/nm. The results are shown in Table 1. (Trial simulations showed that for pure PLPC bilayers this field strength leads to formation of a pore — a membrane-spanning column of water approximately 1 nm diameter surrounded by phospholipid head groups [27] — in less than 25 ns in at least one of three independent trajectories.) Increasing the oxidized lipid content decreases the bilayer thickness, increases the average area per lipid, and reduces the time to poration in an external electric field.

Additional 25 ns simulations with systems containing 50% oxPLPC show that even when the field is reduced far below the minimal porating value for pure PLPC, the oxidized bilayers still form pores, and that membranes containing 12-al porate more readily than 13-tc bilayers (Table 2). Although 12-al bilayers have a slightly smaller area per lipid (which might be expected to restrict the entry of water into the membrane interior), they are thinner than 13-tc bilayers, which lowers the energy barrier for water penetration. In addition, the 12-al aldehyde group spends more time in intermediate locations between the aqueous interface and the low dielectric membrane mid-plane than is the case for the 13-tc hydroperoxy oxygens (Figure 1). As we demonstrate below, the partially hydrated aldehyde group facilitates formation of the membrane-spanning water column that marks the initiation of poration.

**Table 2:** Effect of electric field magnitude on electropore formation time in 50% oxPLPC:PLPC bilayers.

	Mean Poration Time (ns)			
	150 mV/nm	200 mV/nm	250 mV/nm	300 mV/nm
**12-al**	10.4	5.7	3.7	3.8
**13-tc**	>25	14.1	10.2	7.3

Field-dependent poration occurs in oxPLPC:PLPC bilayers even at fields where no poration is observed in pure PLPC membranes during 25 ns simulations. 12-al:PLPC bilayers porate more readily than 13-tc:PLPC bilayers. Systems contain 2880 water molecules, 36 PLPC, and 36 oxPLPC (12-al or 13-tc).

#### Pore formation occurs at local concentrations of oxidized lipids

**Figure 2:**
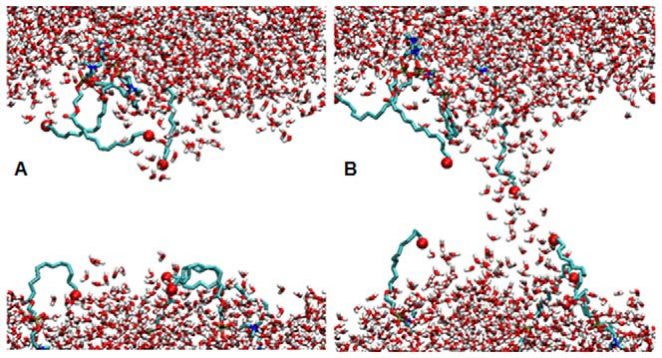
Poration of an oxidized phospholipid bilayer. Snapshots of a PLPC system with an 11% concentration of 12-al oxPLPC before (a) and after (b) an electropore is formed (separated by about 2 ns.) Only water (small red and white "v's") and the head groups and *sn-2* tails of the oxPLPC molecules are shown. The large red spheres near the ends of the tails are aldehyde oxygens, which appear to facilitate the entry of water into the bilayer interior. Dimensions of the simulation box are approximately 5 nm×5 nm×7 nm.

Because the initial steps leading to electroporation involve the intrusion of water into the bilayer interior, and because bilayers containing oxidized species are more permeable to water, we expected that the site of pore initiation in our simulations might be specifically associated with single or aggregated oxPLPC molecules. This is what we observe in every case. The snapshots in Figure 2 are frames taken from a representative simulation (11% 12-al:PLPC in a 360 mV/nm electric field) just before the appearance of a membrane spanning water column (Figure 2a) and just after a pore has formed (Figure 2b), showing clearly the association of the intruding water with the oxidized residues. The initial water defect in bilayers containing 12-al or 13-tc is always associated with the aldehyde or peroxy oxygens in the oxidized tail.

**Figure 3:**
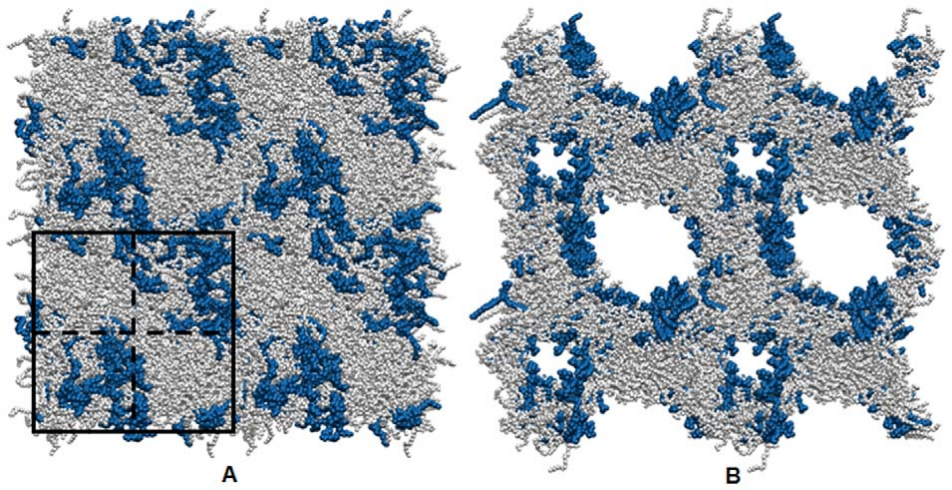
Quilted PLPC:oxPLPC bilayer. The simulated system, bounded by the black square in panel A, is divided (dashed lines) into two regions of approximately 100% PLPC (light gray) and two regions of approximately 50% oxPLPC (12-al) (dark blue) and 50% PLPC, as described in the text. To show more clearly where poration occurs after application of an external electric field, copies of the simulated system are tiled to make a periodic 2×2 system (approximately 10 nm×10 nm×7 nm). Preferential electroporation of the PLPC bilayer in regions of high oxidized lipid content is demonstrated in panel B, which shows the system 1 ns after applying a 360 mV/nm field normal to the bilayer. The bilayer is in the plane of the page.

To visualize this more clearly we created “quilted” PLPC bilayers, with some sections containing 50% oxPLPC and others containing only PLPC, and subjected them to a porating electric field (Figure 3). In all of our simulations electropores invariably form in immediate association with one or more oxidized lipids.

### Observations with Living Cells

To determine whether these simulations are indicative of the responses of living cells, we treated Jurkat T lymphoblasts with sublethal doses of peroxidizing agents (hydrogen peroxide and ferrous sulfate) and then monitored the influx of the fluorescent dye YO-PRO-1, a sensitive indicator of membrane permeabilization [17], after exposure to ultra-short (30 ns), high-intensity (3 MV/m) electric pulses. Conditions for the peroxidation treatment were developed by maximizing the green∶red fluorescence emission ratio from the membrane-staining fluorescent dye C11-BODIPY^581/591^ [43] while at the same time minimizing changes in cell morphology and membrane integrity.

**Figure 4:**
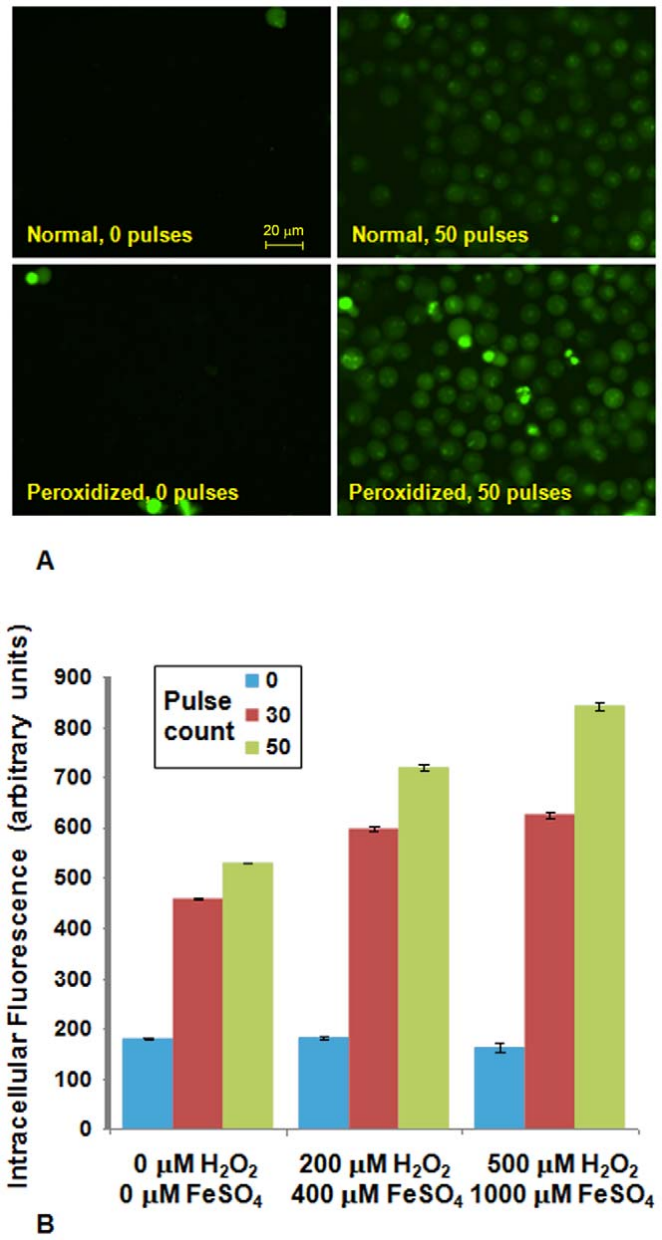
Electropermeabilization enhancement by treatment with peroxidation agents. (A). Fluorescence images of Jurkat cells showing pulse-induced (30 ns, 3 MV/m, 50 Hz) YO-PRO-1 influx into control and peroxidized cells after 10 min exposure to 500 µM H_2_O_2_+1000 µM FeSO_4_. (B). Integrated YO-PRO-1 fluorescence intensity from more than 300 individual cells from three independent experiments for each condition. Error bars are standard error of the mean.

Results are shown in Figure 4. Pulse-induced YO-PRO-1 uptake in peroxidized Jurkat cells is significantly higher than in non-oxidized cells, as predicted by the simulations.

In a parallel set of experiments with calcein-loaded DC-3F (Chinese hamster lung fibroblast) cells, we monitored the decrease in intracellular calcein fluorescence caused by two kinds of permeabilizing electric pulses: ultra-short (10 ns), high-intensity (2.5 MV/m) nanoelectropulses; and long (100 µs), low-field (50 kV/m) pulses typical of those used in electrotransfection and electroporation protocols. This method for detecting permeabilization, which takes advantage of the outward flux of normally impermeant calcein molecules across a steep concentration gradient and into the large reservoir represented by the external medium may be more sensitive than standard procedures for measuring electroporation, which rely on the influx of dye molecules across a decreasing concentration gradient into the relatively small intracellular volume.

**Figure 5:**
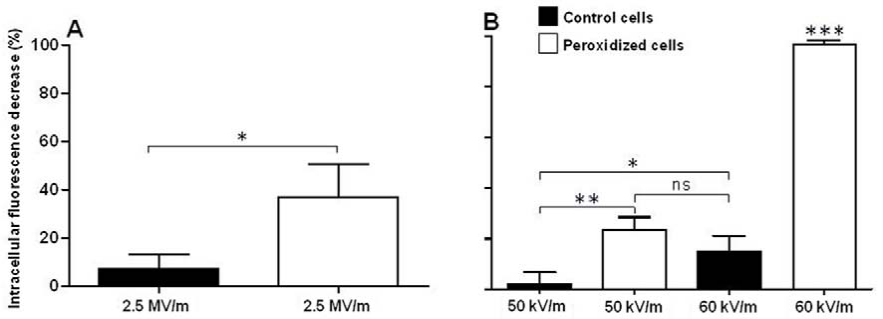
Enhanced electropermeabilization of peroxidized cells with both ultra-short and conventional electric pulse treatments. (A). The fluorescence of calcein-loaded DC-3F (Chinese hamster lung fibroblast) cells exposed to 1000 10 ns, 2.5 MV/m pulses with a 10 Hz repetition rate decreases after 10 min, indicating membrane permeabilization. The effect is much greater in peroxidized cells than in untreated control cells. (B). Peroxidized cells treated with a single 100 µs, 50 kV/m pulse show a similar increased susceptibility to electropermeabilization. Increasing the 100 µs pulse amplitude to 60 kV/m results in significant permeabilization of control cells, indicating that these doses are near the threshold for a detectable response under these conditions. Peroxidized cells treated with a single 100 µs, 60 kV/m pulse show a strong decrease of fluorescence, indicating substantial membrane permeabilization. Bars are the mean, and error bars are the standard deviation. * p<0.05; ** p<0.01; *** p<0.001.

The loss of fluorescence from calcein-loaded DC-3F cells measured 10 minutes after exposure to one thousand 10 ns, 2.5 MV/m pulses delivered at 10 Hz is much greater for peroxidized cells than for the controls, consistent with the Jurkat cell results. The same is true for the response to a single 100 µs, 50 kV/m pulse (Figure 5). Figure 5 also shows that increasing the pulse amplitude to 60 kV/m produces significant permeabilization of untreated cells, indicating that the 100 µs, 50 kV/m dose is near the threshold for detectable permeabilization for these cells, and that membrane peroxidization reduces the value of this threshold.

## Discussion

Molecular dynamics simulations of lipid bilayers and laboratory studies of model membranes and cells in electric fields are consistent with the stochastic pore hypothesis for electropermeabilization [44]–[47]. Here we have shown that it is possible to bias pore formation at the molecular level by oxidatively modifying the properties of the membrane in situ, so that we have now a new method for lowering the energy barrier to poration and a mechanism that might be used to localize where in the membrane poration occurs. This demonstration of the increased sensitivity of oxidatively damaged cells to electropermeabilization has practical implications for the laboratory and the clinic.

### Electrotransfection and Electropermeabilization Enhancement with Peroxidizing Agents

The results suggest that an appropriately controlled peroxidizing regimen, that is, one which enhances permeabilization without significantly affecting viability, might increase the efficiency of electrotransfection protocols either indirectly by enabling the use of lower porating voltages (higher voltages are associated with lower cell survival rates) or directly by increasing the amount of genetic material that enters the cells for a given series of electrical pulses. Alternatively, a reducing environment would be expected to protect cells against electropermeabilization. Extending this line of thinking, electrochemotherapy and direct ablation and killing of tumor cells using electrical pulse therapy may likewise be enhanced by procedures which promote oxidative stress in the tumor tissue before pulse delivery — and inhibited when the environment in or around the tumor is reducing [48]. In any mixed population of cells, the different native sensitivities of various cell types to permeabilizing electric fields [49]–[51] and oxidative stress might be exploited by adjusting electrical, physical, and chemical parameters to selectively transfect subsets of cells, in vitro and in vivo.

### Effects of Cell Handling Procedures on Electroporation Efficiency

Because the simulations show the molecular-scale localization of electroporation enhancement by oxidized membrane lipids, it is not unreasonable to infer that improper handling of cell suspensions, resulting in uncontrolled and unmonitored degrees of oxidative stress, may account for inconsistent and variable results of electrotransfection and other methods dependent on reversible electropermeabilization. Cells maintained at high concentrations in rich medium, or for long periods in non-nutritive buffers may be unexpectedly and unpredictably sensitive to porating electric pulses. Likewise, adding reducing agents to cell suspensions to protect labile compounds or the cells themselves [52] may unintentionally result in decreased electropermeabilization and electrotransfection yields. Investigations of these possibilities, although they will of necessity be protocol- and cell type-specific, have the potential to lead to more consistent and more efficient membrane permeabilization procedures.

### Oxidative Stress and Apoptosis

The relationship between oxidative stress and electropermeabilization sensitivity may have another dimension. It is known that exposure to nanosecond, megavolt-per-meter electric pulses can not only permeabilize membranes [17], [53] but also induce apoptosis [50], [54]. The role of reactive oxygen species (ROS) in apoptosis has been investigated [55]–[57], and we report here that oxidative stress also modulates electropermeabilization. A more extensive analysis of oxidative stress and short and long pulse electroporation may lead to a more sophisticated approach to controlling electrotransfection yield, including both the efficiency of the incorporation of the genetic material and the subsequent viability of the cells, which may be decreased by pulse-induced apoptosis. Similar considerations apply to potential improvements in electroporation protocols. Thus the knowledge gained from an exploration of the interplay between nanosecond membrane permeabilization [17], [58] and conventional electroporation [59] in the presence of excess reactive oxygen species and the initiation of apoptosis may result in improved electroporation and electrotransfection procedures, with benefits from cell science to cancer therapeutics.

### Permeabilizing Defects and Membrane Boundaries

Facilitation of electric field-driven water defect formation by the aldehyde and hydroperoxy oxygens of the oxPLPC species reported here may in fact be a relatively simple example of a more general tendency for water intrusion, permeabilization, and other membrane restructuring events to occur at membrane phase or domain boundaries, especially where these discontinuities have charged or hydrophilic components that extend into the membrane interior [44], [60]–[62]. In this context it will no doubt prove instructive to examine the combined effects of lipid peroxidation and cholesterol on membrane electropermeabilization. Adding cholesterol to a bilayer is likely to render the membrane more difficult to electropermeabilize [63], but the consequences of incorporating peroxidized lipids, which promote the formation of cholesterol domains and lipid rafts [64], [65], into the system are difficult to predict. Increasing computational power makes it possible now to address these more complex (and more realistic) systems with an approach that ties together atomically detailed simulations and experimental cell biology.

## Materials and Methods

### Cell Lines and Culture Conditions

Jurkat T lymphoblasts (ATCC TIB-152) were grown in RPMI 1640 medium (Mediatech) containing 10% heat-inactivated fetal bovine serum (FBS; Mediatech), 2 mM L-glutamine (Invitrogen), 50 units/mL penicillin (Gibco), and 50 µg/mL streptomycin (Gibco) at 37°C in a humidified, 5% carbon dioxide atmosphere. DC-3F adherent cells (Chinese hamster lung fibroblast cells) were grown in Minimum Essential Medium (Invitrogen, France) containing 10% heat-inactivated FBS (Invitrogen), 500 U/ml penicillin, 500 µg/ml streptomycin (Invitrogen) at 37°C in a humidified, 5% carbon dioxide atmosphere.

### Cell Preparation

Jurkat cells were exposed to peroxidizing conditions — H_2_O_2_ (200 and 500 µM) and FeSO_4_ (400 and 1000 µM) in RPMI 1640 — for 15 minutes before pulse exposure. After peroxidation, 5 µM YO-PRO-1 (Molecular Probes, Invitrogen) was added as a permeabilization indicator, and the cells were immediately pulsed. DC-3F cells were incubated with 1 µM calcein-AM (Sigma-Aldrich, France) in culture medium for 1 hour at 37°C, then rinsed with PBS, trypsinized, and centrifuged at 1000 rpm for 10 minutes. For peroxidation, DC-3F cells were incubated with H_2_O_2_ and FeSO_4_ (each 1000 µM for long pulse experiments, 1500 µM for short pulse experiments) for 1 hour at 37°C, rinsed with PBS and centrifuged at 1000 rpm for 10 minutes. The pellets were suspended in sucrose buffer (250 mM sucrose, 10 mM Tris, 1 mM MgCl_2_ — Sigma), pH 7, containing 2% low-melting agarose (Tebu-Bio, France) at about 50 000 cells/mL. Cell suspensions were kept at 37°C until electric pulse exposure.

### Pulse Generator and Pulse Exposures

For the Jurkat cell experiments 30 ns, 3 MV/m pulses were delivered at a 50 Hz repetition rate to cell suspensions in commercial electroporation cuvettes (VWR) with a 1 mm electrode spacing in ambient atmosphere at room temperature from a USC pulse generator based on a magnetic compression, diode opening-switch architecture [66]. For the DC-3F experiments the cell suspension was placed within the 2 mm gap between two copper electrodes fixed to a microscope slide. Cells were exposed to 1 long pulse (100 µs, 50 or 60 kV/m) delivered by a micropulse generator (Cliniporator, IGEA, Italy) or to 1000 short pulses (10 ns, 2.5 MV/m, 10 Hz) delivered by a FID Technology (Russia) pulse generator.

### Fluorescence Microscopy and Microphotometry

Jurkat cell images were captured and analyzed with a Zeiss AxioCam MRm and AxioVision 3.1 software (Carl Zeiss Goettingen, Germany) on a Zeiss Axiovert 200M epifluorescence microscope. Intracellular YO-PRO-1 (λ_ex_ = 480 nm, λ_em_ = 535 nm) fluorescence was used as an indicator of membrane permeabilization [67], [68]. Membrane lipid peroxidation was monitored qualitatively with the fluorescent dye C11-BODIPY^581/591^. Upon oxidation, the dye shifts from a red-emitting form (595 nm) to a green-emitting form (520 nm) [69]. Cells were incubated in RPMI 1640 medium containing 4 µM C11-BODIPY^581/591^ for 30 minutes at 37°C before exposure to peroxidizing reagents. Images of calcein-loaded DC-3F cells were taken immediately before and 10 min after pulse delivery at λ_ex_ = 496 and λ_em_ = 516 nm, using a Zeiss AxioCam HRC camera coupled to a Zeiss Axiovert S100 microscope. Intracellular fluorescence was averaged over more than 300 cells. Each experiment was repeated three times. For each repetition of the calcein efflux experiments the intracellular fluorescence (F) was measured on the same cells (each time, more than 300 cells) before (F_b_) and 10 minutes (F_10_) after the pulses delivery. The normalized decrease in fluorescence (F_b_−F_10_)/F_b_ was averaged (+/− SD) over the 3 repeats. * = p<0.05; ** = p<0.01; NS = no statistically significant difference.

### Molecular Dynamics Simulations

All simulations were performed using the GROMACS set of programs version 3.3.1 [70] on the University of Southern California High Performance Computing and Communications Linux cluster (http://www.usc.edu/hpcc/). Lipid parameters were derived from OPLS, united-atom parameters [71] modified for PLPC and oxPLPC [44]. We used the Simple Point Charge (SPC) model [72] for water. Systems were coupled to a temperature bath at 310 K with a relaxation time of 0.1 ps and a pressure bath at 1 bar with a relaxation time of 1 ps, each using a weak coupling algorithm [73]. Pressure was scaled semi-isotropically with a compressibility of 4.5×10^−5^ bar^−1^ in the plane of the membrane and 4.5×10^-5^ bar^−1^ perpendicular to the membrane. Bond lengths were constrained using LINCS [74] for lipids and SETTLE [75] for water. Short-range electrostatics and Lennard-Jones interactions were cut off at 1.0 nm. Long-range electrostatics were calculated by the PME algorithm [76] using fast Fourier transforms and conductive boundary conditions. Reciprocal-space interactions were evaluated on a 0.12 nm grid with fourth order B-spline interpolation. The parameter ewald_rtol, which controls the relative error for the Ewald sum in the direct and reciprocal space, was set to 10^−5^. Periodic boundary conditions were employed to mitigate system size effects.

### Electroporation Simulations

To determine a baseline porating electric field [77], simulations of equilibrated (constant area per lipid), fully hydrated (40 water/lipid) PLPC bilayer systems containing 72 lipid molecules and 2880 water molecules [42] were run with applied electric fields ranging from 300 to 500 mV/nm. The lowest field which results in pore formation within 25 ns in at least one of three parallel PLPC simulations is 360 mV/nm, and that value was used for comparisons of pore formation times in oxidized (PLPC:oxPLPC) and non-oxidized (PLPC) systems. Oxidized lipid systems included 8 (11.1%), 18 (25%), or 36 (50%) randomly distributed molecules of oxPLPC, either 12-al or 13-tc [42].

Electroporation times were calculated by measuring at each time step the number of phosphorus atom groups in a system, where a group is a cluster of phosphorus atoms each no more than 1.2 nm from another phosphorus atom [78]. The two phosphorus atom groups in an intact bilayer (one in each of the two leaflets) merge when the bilayer interior is bridged by a hydrophilic pore. We call the time at which this occurs the electroporation time. In some simulations the merged groups split again, in each case in less than 400 ps after merger. This was not considered pore formation.

### Images

Molecular graphics images were generated with Visual Molecular Dynamics (VMD) [79].
